# Ectodermal Dysplasia: A Case Report

**DOI:** 10.5005/jp-journals-10005-1124

**Published:** 2011-04-15

**Authors:** Vinay K Srivastava

**Affiliations:** Professor and Head, Department of Pedodontics and Preventive Dentistry, Saraswati Dental College, Lucknow, Uttar Pradesh, India

**Keywords:** Midface hypoplasia, Double lip, Partial anodontia.

## Abstract

Ectodermal dysplasia is a hereditary disease characterized by dysplasia of tissues of ectodermal origin. The incidence of ectodermal dysplasia is rare (1 in 100,000 birth). This case report discusses the features, classification and prosthetic treatment plan (upper partial denture and lower complete denture for upper partial and lower complete edentulous arches respectively). This treatment plan would be able to provide psychological and functional boost to the sufferer.

## INTRODUCTION

Ectodermal dysplasia is a heterogeneous group of inherited disorders, resulting from the abnormal development of two or more tissues at a time, derived from embryonic ectoderm. Ectodermal dysplasia is a congenital, diffuse and nonprogressive disease. The pure ectodermal dysplasia is manifested by defects in ectodermal structures alone, while ectodermal dysplasia syndrome is a combination of ectodermal defects in association with other anomalies. The most common ectodermal dysplasia is X-linked recessive hypohidrotic ectodermal dysplasia and hidrotic ectodermal dysplasia.

## CASE REPORT

**Figs 1A to F:**
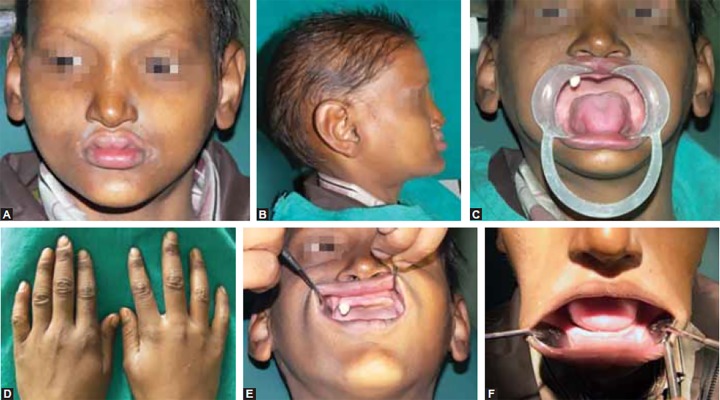
(A) Dry skin, everted upper double lip and scanty eyebrows, (B) large low set ears with midface hypoplasia, (C) partial anodontia, (D) thin and brittle nails, (E and F) upper and lower arch with reduced vertical height of alveolar bone respectively

An 11-year-old male of Indian origin visited the Department of Pedodontics and Preventive Dentistry, Saraswati Dental College, Lucknow, India, with the chief complaint of absence of teeth in his oral cavity since childhood and a single permanent tooth erupted in the oral cavity (Figs 1C and E) with dryness of mouth. There was no history of birth complications during his delivery, and no other live family member presented similar condition. On clinical examination, dry and scaly skin with slightly elevation of temperature was observed. Hair examination revealed fine sparse, luster-less appearance and very thin eyebrows. Eye examination showed dryness of cornea and decreased lubrication/tearing. Fingers’ examination revealed normal shaped fingers with thin, brittle nails (Fig. 1D). Ear examination revealed large low set ears with midface hypoplasia gives an older look as compared with those of his age with a normal intelligence. Patient had typical facies which was characterized by saddle nose, thick everted lips with accentuated double lip. Intraoral examination revealed dry mucous membrane with single conical tooth erupted in anterior right region of the maxilla and reduced vertical heights of both the arches were of considerable significance (Figs 1E and F).

## CAUSES

Ectodermal dysplasia results from developmental defect of embryonic ectodermal structures. The genetic defects responsible for approximately 30 of the ectodermal dysplasia have been identified.^2-4^

1. X-linked recessive hypohidrotic ectodermal dysplasia (EDA or Christ-siemens-Touraine syndrome) is caused by mutation in EDA, which encodes for the ectodysplastin protein, a soluble ligand that activates the NF-kappa B and JNK- fos/c-jun signaling pathways^5,6^
2. Hidrotic ectodermal dysplasia, which is an autosomal dominant disorder, is caused by mutation in GJB, which encodes for connexin 30, a component of intercellular gap junction^7^
3. Autosomal dominant and autosomal recessive hypohidrotic ectodermal dysplasia are caused by mutation in the DL gene, which encodes for the EDA recepter.^8^

## DISCUSSION

Freire-Maia and Pinheiro proposed the first classification system of ectodermal dysplasia in 1982.^1^ They classified ectodermal dysplasia into different subgroups according to the presence or absence of (1) hair anomalies or trichodysplasia, (2) dental anomalies, (3) nail abnormalities or onychodys-plasia, (4) eccrine gland dysfunction or dyshidrosis. As in the above-mentioned case report, all features of ectodermal dysplasia classification were present; like scanty hair, partial anodontia, thin-brittle nails, dry mouth as well as dry cornea. The above classification can be modified with addition of a fifth subgroup having features of subgroups 1 to 4.

The ectodermal dysplasia were also classified into either group A disorder which were manifested by defect in at least 2 of the 4 classical ectodermal structure as defined above, with or without other defect and group B disorders which were manifested by a defect in one classical ectodermal structure (1-4 from above) in combination with (5) a defect in any one of the ectodermal structure (ear, lip; Figs 1A and B) but in our case report all classical ectodermal defects were found with defect in upper lip (accentuated double lip, Figs 1A) which suggest that this ectodermal dysplasia will be included in group B disorders.

Medical care of ectodermal dysplasia depends on which ectodermal structure is involved. In the above case report, the temperature of the body was slightly elevated so it would be advisable to have frequent consumption of cool liquids to maintain adequate hydration and thermoregulation. For a patient with dental defects, early dental evaluation and intervention is advised and encouragement for routine dental hygiene is done. In above case report, upper partial denture and lower complete denture was advised. After construction of dentures patient was educated for adjustments and reconstruction of dentures at different stages of growth and advised that dental implants may eventually be required. Patients with xerostomia and reduced lacrimation may benefit from artificial saliva and tears respectively. The defect of lip can be corrected by cheiloplasty to improve esthetics. General dentist can provide regular preventive dental care and restorative services as indicated. Patient is advised to take consultation with genetic counselor to find out the diagnosis and genetic analysis. No dietary restrictions are indicated. The prognosis of the ectodermal dysplasia is very good and the life span of the patient is usually normal except for case of ectodermal dysplasia with immunodeficiency.
